# Distributed Temperature and Strain Discrimination with Stimulated Brillouin Scattering and Rayleigh Backscatter in an Optical Fiber

**DOI:** 10.3390/s130201836

**Published:** 2013-01-31

**Authors:** Da-Peng Zhou, Wenhai Li, Liang Chen, Xiaoyi Bao

**Affiliations:** Department of Physics, University of Ottawa, Ottawa, ON K1N 6N5, Canada; E-Mails: wenhai.li@uottawa.ca (W.L.); liang.chen@uottawa.ca (L.C.); xiaoyi.bao@uottawa.ca (X.B.)

**Keywords:** optical fiber sensors, temperature and strain discrimination, stimulated Brillouin scattering, Rayleigh scattering

## Abstract

A distributed optical fiber sensor with the capability of simultaneously measuring temperature and strain is proposed using a large effective area non-zero dispersion shifted fiber (LEAF) with sub-meter spatial resolution. The Brillouin frequency shift is measured using Brillouin optical time-domain analysis (BOTDA) with differential pulse-width pair technique, while the spectrum shift of the Rayleigh backscatter is measured using optical frequency-domain reflectometry (OFDR). These shifts are the functions of both temperature and strain, and can be used as two independent parameters for the discrimination of temperature and strain. A 92 m measurable range with the spatial resolution of 50 cm is demonstrated experimentally, and accuracies of ±1.2 °C in temperature and ±15 με in strain could be achieved.

## 1. Introduction

Optical fiber sensors (OFSs) have attracted intensive research interest around the World for several decades. They have already shown a superior advantage over their conventional electrical counterparts because of their distributed capabilities. A fully distributed OFS is usually operated by measuring the surrounding environment changes along the length of the sensing fiber. Several mechanisms have been successfully applied to fulfill this kind of measurement, such as Brillouin scattering, Raman scattering, as well as Rayleigh scattering. In terms of approaches, optical time-domain reflectometry (OTDR) and optical frequency-domain reflectometry (OFDR) have found their way to meet various practical needs. On the other hand, among a large amount of physical and chemical parameters which OFSs could measure, temperature and strain are the most widely studied, since many applications require accurate measurement of these two parameters, such as monitoring the health of large structures for their conditions, detecting pipeline buckling or leaking, and bridge deformation.

An OFS is sensitive to both strain and temperature, normally making a change in temperature indistinguishable from a change in strain. This cross sensitivity, which exists in almost all the OFSs, including distributed ones, would introduce errors when monitoring strain. In a Brillouin scattering based distributed sensor, as the Brillouin frequency is sensitive to both temperature and strain, and the Brillouin power in single-mode fiber (SMF) is also sensitive to temperature and strain, so Brillouin power and frequency can be used for simultaneous temperature and strain measurement [1]; however, due to the power fluctuation and random change of state of polarization in the measurement, the spatial resolution has been limited to 3 m, with temperature accuracy of 4 °C and strain resolution of 180 με for SMF28. This result is much worse than that achievable in single parameter sensing with the BOTDA technique. Alternative techniques have been proposed: the use of two different kinds of fibers with different refractive indices in the fiber core [2], which requires two fibers and reduces the sensing range by 1/2; two acoustic resonance peaks at different orders of Brillouin gain spectrum are monitored [3,4] in large effective area non-zero dispersion shifted fiber (LEAF) and photonic crystal fiber (PCF); the power of the Brillouin scattered light and Brillouin frequency or the Brillouin frequency and Brillouin spectrum bandwidth are measured at the same time in polarization-maintaining fiber (PMF) [5]; both the spontaneous Raman and Brillouin backscattered signals are resolved and the anti-Stokes Raman signal could permit the determination of the temperature [6–8]; birefringence in a PMF and Brillouin frequency are determined simultaneously [9–12]. Generally speaking, in special fibers, sub-meter spatial resolution can be achieved for simultaneous temperature and strain measurement; however the limited sensing length, launching and splicing difficulty with SMF [4,5,9–12] make those fibers hard to be used in practical applications. In the meantime, in SMFs (SMF28e and LEAF), the reported spatial resolution is on the order of meters [1,3]. Because of small differences in the temperature and strain coefficients of the individual Brillouin peaks in LEAF, it is very difficult to detect small Brillouin frequency differences over 10 GHz frequencies; this has limited the temperature and strain accuracy: 5 °C and a strain resolution of 60 με and a spatial resolution of 2 m [3]. The reported hybrid Raman-Brillouin sensor has a relatively large spatial resolution (tens of meters) [6–8].

For the OFDR technique, temperature and strain discrimination could be achieved in a PMF by measuring the autocorrelation and cross-correlation shifts of the Rayleigh backscatter data [13] with the sensing length of 8 m demonstrated. PMF requires special launching and delaunching techniques, which makes sensor system complicated.

In this work, we propose to measure temperature and strain simultaneously with stimulated Brillouin scattering and Rayleigh backscatter in a single-mode fiber. Distributed Brillouin frequency shift (BFS) is measured by differential pulse-width pair Brillouin optical time-domain analysis (DPP-BOTDA) [14,15]. By subtracting the time-domain waveform at the same scanned Brillouin frequency obtained from light pulses with different pulse widths, the differential Brillouin gain spectrum at each fiber position is obtained so that the spatial resolution is greatly enhanced. Distributed Rayleigh backscatter spectral shift (RBSS) is obtained through OFDR technique [16–20], which takes the measurement of complex Rayleigh backscatter pattern of the testing fiber. Any ambient temperature or strain variations can change the scatter pattern. By calculating the cross-correlation of the Rayleigh backscatter spectral data from each small fiber section of varied temperature or strain states with respect to unchanged states, distributed RBSS can be obtained. A well-conditioned mapping matrix could be achieved for temperature and strain discrimination. After a pair of BFS and RBSS has been measured along the sensing fiber, distributed temperature and strain information could be determined. It is worth mentioning that a hybrid Brillouin-Rayleigh system was reported recently [21], and the Rayleigh responses are measured by tunable wavelength coherent OTDR. The technique measures the jagged appearance of Rayleigh backscatter traces with a tunable narrow linewidth light source whose frequency is precisely controlled [22]. The coherent OTDR traces are measured and recorded repeatedly while the laser frequency is changed step by step. Therefore, Rayleigh backscatter spectrum which is sensitive to temperature or strain can be obtained distributedly along the sensing fiber. This technique has advantages of long measurement range and high temperature or strain resolution. The spatial resolution is determined by the pulse width. However, the entire Rayleigh spectrum can be measured by obtaining a huge number of coherent OTDR traces over a large bandwidth (e.g., 5 THz) with small frequency step which is quite time consuming (a few of minutes to several hours) [21,22]. On the other hand, the OFDR technique measures complex Rayleigh backscatter patterns that makes it feasible for distributed temperature or strain measurement with very high spatial resolution. The technique has a fast measurement time usually on the order of seconds, but with a limited measurement range. The total sensing range demonstrated in this work is about 92 m with a spatial resolution of 50 cm with temperature and strain accuracies of ±1.2 °C and ±15 με. The measurable range is limited by our current OFDR setup, which could be extended to at least several hundreds of meters (see [23], over 600 m measurable range achieved with OFDR). The spatial resolution can be improved to centimeter range considering recent achievement in Brillouin sensing technique [21,24].

## 2. Experimental Setup and Operation Principle

**Figure 1.**
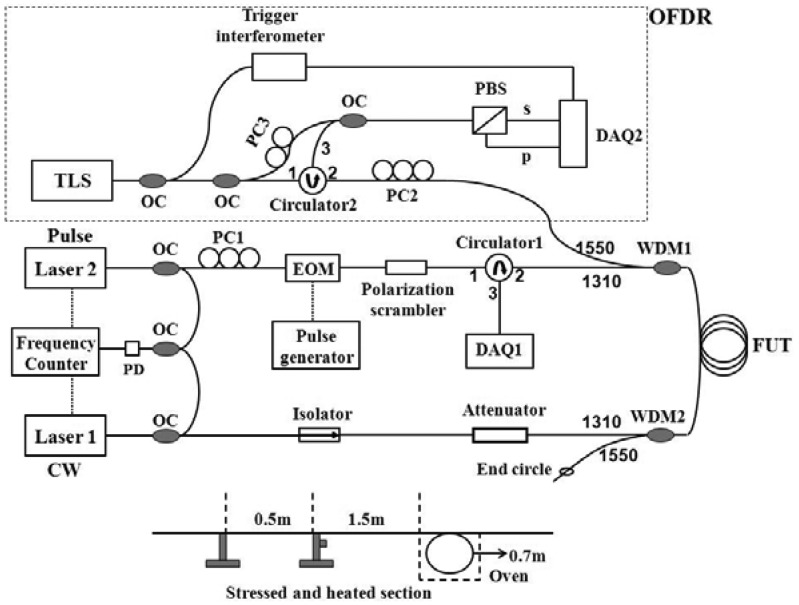
Experiment Setup. OFDR: optical frequency-domain reflectometry; TLS: tunable laser source; DAQ: data acquisition; EOM: electro-optic modulator; OC: optical coupler; PC: polarization controller; PD: photodetector; PBS: polarization beam splitter; WDM: wavelength division multiplexer; FUT: fiber under test. Bottom inset shows the sensing fiber section, where a 0.5 m long fiber could be applied axial strain by attaching the fiber on the translation stage, and a 0.7 m section is placed in the oven for varying temperature; the two sections are separated by about 1.5 m.

The experimental setup is shown in Figure 1. Two Nd:YAG lasers operating at 1,319 nm are used as laser sources for the BOTDA system [14]. Their frequency difference is locked by a phase-locking loop in a frequency counter via a photodetector (PD). The electro-optic modulator (EOM) driven by a pulse generator is used to generate optical pulses. Pulsed Stokes beam is injected into a sensing fiber and experiences Brillouin amplification at the expense of the counter-propagating continuous wave (CW) pump beam. The resultant power drops in the pump beam are measured while the frequency difference between the lasers is scanned across Brillouin spectrum. With the use of a pair of the pulsed beam to perform the DPP-BOTDA processing [14], the distributed BFS of the fiber could be determined from the differential spectrum and is used to calculate the strain or the temperature along the sensing fiber. The spatial resolution is determined by the pulse width difference.

The OFDR configuration consists of a tunable laser source (TLS; Agilent 81980A) operating around 1,550 nm with a continuous sweep mode. Since the polarization state of the scattered signal is arbitrary, we adopt the polarization diversity detection technique. When the laser is tuned, the interference signals obtained from the combination of the Rayleigh scattering signal and the local laser beam is split by a polarization beam splitter (PBS); the resultant “s” and “p” components are then received and digitized as a function of the TLS frequency by a two-channel acquisition. Fourier transform is then used to convert these frequency data into time-domain data; after performing a vector sum of the transformed “s” and “p” components [18], the Rayleigh backscatter pattern *versus* the length of the fiber under test (FUT) could be obtained. Since the data acquisition and processing are performed in a discrete manner, the Rayleigh backscatter data has a step size, Δ*L*, which is determined by the scan range of the TLS, Δ*λ*_TLS_, in the measurement:

(1) $$\Delta L\approx \frac{\lambda^{2}}{2n_{g}\Delta \lambda_{\text{TLS}}}$$

where *n_g_* is the group index of the FUT; *λ* is the central operating wavelength. The distributed temperature or strain information could be obtained by calculating the cross-correlation of the Rayleigh backscatter spectrum for a certain fiber section in the temperature or strain varied states with unchanged states. The length of this section determines the spatial resolution of the OFDR for temperature or strain measurement. Normally, there is a trade-off between temperature or strain resolution and the spatial resolution [17]. The signal-to-noise ratio of the cross-correlation could be improved if a longer fiber section is chosen which results in poorer spatial resolution but a better strain or temperature resolution. This spatial resolution should be properly chosen according to the system's performance as well as the practical requirements. The trigger interferometer is an auxiliary Mach-Zehnder interferometer which is commonly used in OFDR systems to remove laser tuning errors from the data [16–20]. Moreover, the maximum length of the FUT, *L*_max_ is determined by the differential delay in the trigger interferometer using the Nyquist sampling criteria by [19]:

(2) $$L_{\max}=\frac{c\tau_{g}}{4n_{g}}$$

where *c* is the velocity of light in vacuum; *τ_g_* is the group delay introduced by the trigger interferometer. Although the BFS and RBSS are both sensitive to temperature and strain, their temperature and strain sensitivities are very different, since these two parameters are coming from totally different mechanisms and independent to each other; therefore, complete temperature and strain discrimination could be realized by measuring a pair of BFS and RBSS along a single-mode fiber.

The two systems are combined together with a 1,310/1,550 nm wavelength division multiplexer 1 (WDM1). WDM2 is used to couple the TLS beam out of the fiber loop after it passes through the FUT; otherwise, the Rayleigh backscatter data might be corrupted by the reflection of the BOTDA components such as the attenuator and the isolator. A small fiber circle is made at the end of 1550-nm port of the WDM2 shown in Figure 1 to decrease Fresnel reflection. The distributed temperature and stain information could be measured by operating BOTDA first, and then running OFDR system immediately after making the BOTDA sources standby. After calibrating the temperature and strain coefficients of both the BFS and RBSS, the ambient temperature change, Δ*T*, and the applied strain, *ε*, could be calculated through:

(3) $$\left[\begin{array}{c} \Delta T\\ \varepsilon \end{array}\right]=\frac{1}{D}\left[\begin{array}{cc} K_{\varepsilon ,\text{RBSS}} & -K_{\varepsilon ,\text{BFS}}\\ -K_{T,\text{RBSS}} & K_{T,\text{BFS}} \end{array}\right]\left[\begin{array}{c} \Delta \upsilon_{B}\\ \Delta \lambda \end{array}\right]$$

provided that a pair of BFS, Δ*υ_B_*, and RBSS, Δ*λ*, have been measured. *K_T_*_,BFS_ and *K_T_*_,RBSS_ are the temperature coefficients of BFS and RBSS respectively, while *K_ε_*_,BFS_ and *K_ε_*_,RBSS_ are the corresponding strain coefficients. *D* = *K_ε_*_,RBSS_*K_T_*_,BFS_ − *K_ε_*_,BFS_*K_T_*_,RBSS_. The strain and temperature resolution of the sensor is determined by the condition of the mapping matrix in Equation (3) and the system measurement accuracy. The uncertainty *δ*(Δ*T*) and *δ*(*ε*) could be estimated conveniently by:

(4) $$\left[\begin{array}{c} \delta (\Delta T)\\ \delta (\varepsilon ) \end{array}\right]=\pm \frac{1}{\left|D\right|}\left[\begin{array}{cc} \left|K_{\varepsilon ,\text{RBSS}}\right| & \left|K_{\varepsilon ,\text{BFS}}\right|\\ \left|K_{T,\text{RBSS}}\right| & \left|K_{T,\text{BFS}}\right| \end{array}\right]\left[\begin{array}{c} \delta (\Delta \upsilon_{B})\\ \delta (\Delta \lambda ) \end{array}\right]$$

Where *δ*(Δ*υ_B_*) and *δ*(Δ*λ*) are the measurement uncertainties of the BFS and RBSS, respectively. This provides a convenient way to compare the discrimination capability for different configurations. In our experiment, we intently use LEAF as the sensing fiber showing that using different peaks of BFS may not achieve fine temperature and strain accuracy.

## 3. Experimental Results and Discussion

**Figure 2.**
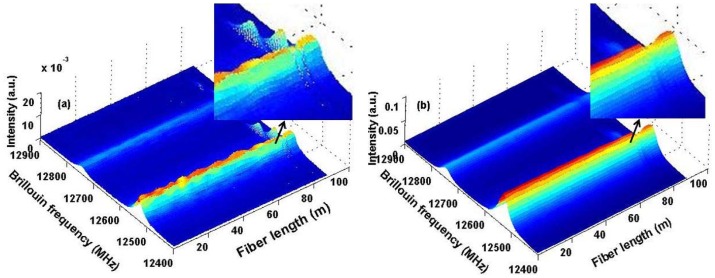
The 3-dimensional graphs of the Brillouin gain spectrum using (**a**) a 45/50 ns pulse pair (**b**) a single 50 ns pulse.

We use a 45/50 ns pulse pair for DPP-BOTDA measurement, determining a 50 cm spatial resolution. The peak power of the optical pulse is approximately 35 mW, and the continuous wave (CW) power from the other source is about 1 mW. The time-domain signals are monitored with a 1 GHz bandwidth AC-coupled PD, and 5,000 averages are taken at each frequency step. The sensing fiber is 92-m long, where near the end of the sensing fiber around 80 m, a section of 0.5 m fiber is used for axial strain measurement by attaching the fiber on a translation stage, and a 0.7 m section is placed in an oven for temperature measurement; the two sections are separated by about 1.5 m as shown in the bottom inset in Figure 1. As an example, we applied strain and increase the temperature to these two fiber sections simultaneously, and the 3-dimensional graph of the differential Brillouin gain spectrum for a 45/50 ns pulse pair is shown in Figure 2(a), while Brillouin gain spectrum for a single 50-ns pulse is shown in Figure 2(b). It is clearly seen that the stressed and heated sections overlap each other due to the spatial resolution of 5 m determined in the single 50 ns pulse case. However, by using a 45/50 pulse pair, the two sections are clearly resolved.

**Figure 3.**
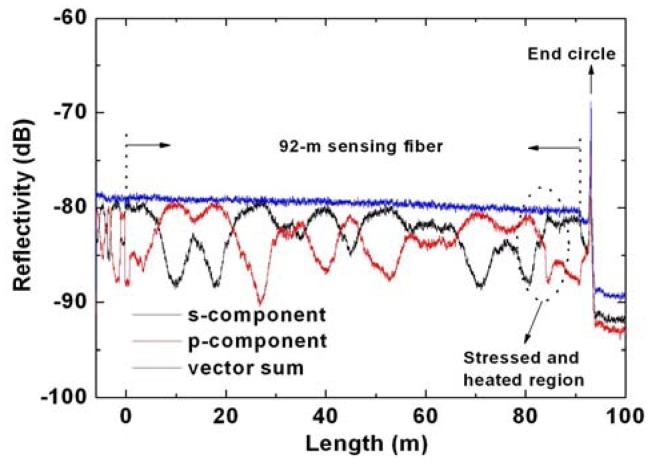
Rayleigh backscatter as a function of fiber length.

For the OFDR measurement, when the TLS is tuned, interference fringes that can be related to the complex reflectivity of the FUT are received at the detectors labeled “s” and “p” via a PBS, triggered by the trigger interferometer. The Rayleigh backscatter as a function of fiber length is obtained through Fourier transform [18], and the results are shown in Figure 3. The blue curve is obtained by vector summing the two Fourier transformed signals of “s” and “p” components. The Rayleigh backscatter data in front of the sensing fiber comes from fiber components of polarization controller 2 (PC2) and WDM1; while near the end circle reflection after the sensing fiber, the Rayleigh backscatter data shown in Figure 3 corresponds to WDM2. The TLS is tuned over 8 nm from 1,545 nm to 1,553 nm, corresponding to a ∼0.1 mm spatial separation of two successive data points by Equation (1). The length of delay line used in the trigger interferometer is about 450 m, corresponding to a maximum of ∼110 m sensing range according to Equation (2). The strain or temperature information could be obtained by measuring the RBSS of the stressed or heated section with respect to the same section when no strain applied or temperature changed through cross-correlation [13,17]. In our experiment, a ∼16 cm fiber section is chosen to perform cross-correlation calculation which corresponds to a 16 cm spatial resolution. There are two reasons for choosing this 16-cm fiber section. One is that it has to be comparable to the spatial resolution determined by DPP-BOTDA; the other one is that it is desirable to be smaller than 50 cm, so that the transition region observed from the DPP-BOTDA could be clearly identified (see Figure 5).

**Figure 4.**
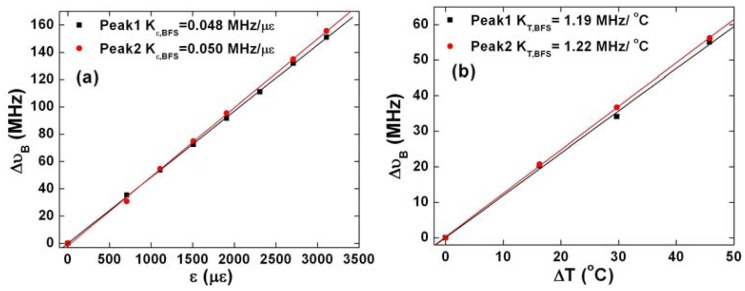

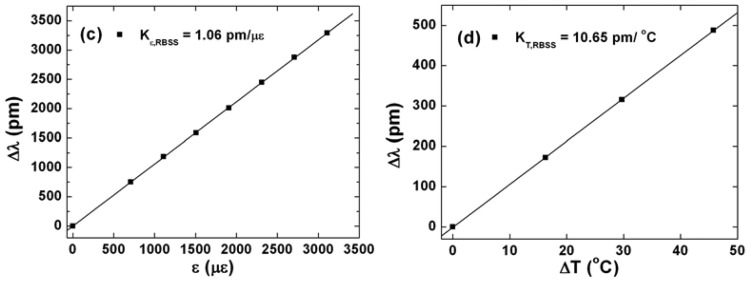
Calibration of (**a**) strain coefficients (**b**) temperature coefficients for both the Brillouin peaks in LEAF (**c**) strain coefficient (**d**) temperature coefficient for RBSS.

Next, we calibrate the temperature and strain coefficients for BFS and RBSS by operating the two systems separately. We measure the BFSs for the first two peaks in LEAF obtaining their strain and temperature coefficients by linearly fitting the experimental data at different strain values and temperatures shown in Figure 4(a,b). When measuring the strain coefficients, we keep the temperature unchanged, and *vice versa*. Both the strain and temperature coefficients of the second peak are slightly larger than those of the first peak. The RBSS coefficients could be obtained in the similar way as shown in Figure 4(c,d). With these coefficients, temperature and strain discrimination could be achieved through Equation (3), provided that a pair of BFS of the first peak and the RBSS has been measured. Repeated experiments show that our measurement uncertainties for BFS of the first peak as well as the RBSS are 0.7 MHz and 3 pm respectively, which could be used to estimate the temperature and strain accuracies by Equation (4) as ±1.2 °C in temperature and ±15 με in strain. We also estimate the performance if the BFSs of the first two peaks in LEAF are used with a measurement uncertainty for the second peak of 1.5 MHz resulting in a temperature and strain accuracies of ±113.8 °C and ±2807 με respectively. Based on this estimation, it is not appropriate to use the two Brillouin peaks in LEAF for distributed temperature and strain discrimination, since the coefficients are very close to each other resulting in an ill-conditioned mapping matrix. This result is in line with reference [3] while large pulse width has been used to offset the temperature and strain uncertainty.

**Figure 5.**
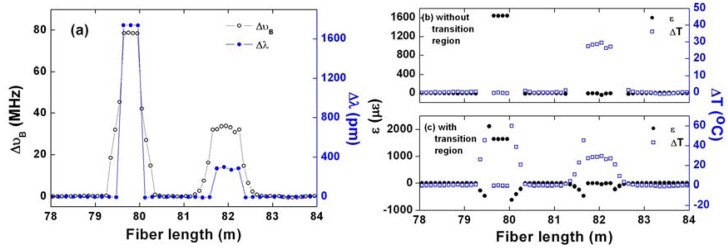
Measured (**a**) BFS of the first Brillouin peak and RBSS. Calculated temperature and strain distribution (**b**) without (**c**) with the transition region as a function of fiber length.

Finally, we investigate the distributed capability. We applied strain of 1,640 με and heat the fiber to 53 °C (room temperature of 25 °C) on two fiber sections simultaneously shown as the bottom inset in Figure 1. At the heated region, both the BOTDA data and RBSS data show small fluctuations primarily due to the non-uniform temperature distribution inside the oven. The BFS of the first peak and the RBSS as a function of fiber length is shown in Figure 5(a). Since we use 1 GHz PD to record Brillouin gain spectrum, the separation of the successive data points corresponds to 10 cm. Due to the finite spatial resolution, transition region shows two-peak spectrum for the BFS measurement, so that the BFS is determined through power weight of intensity of individual peaks [11]. For RBSS measurement, no significant transition effect was observed.

In order to calculate the distribution of the temperature and strain, we re-sample the RBSS data by nearest-neighbor interpolation to obtain the same amount data points with those obtained through DPP-BOTDA. Then, substituting the re-sampled RBSS Δ*λ*, and the BFS Δ*υ_B_*, into Equation (3), the distributed temperature and strain information could be obtained along the sensing fiber. The results of the discrimination of temperature and stain are shown in Figure 5(b,c). Note that, since the DPP-BOTDA has a much larger spatial resolution than that of the OFDR, there are large errors at transition region as shown in Figure 5(c). However, as the stressed or heated region could be well determined by RBSS distribution along the fiber, it is reasonable to identify the transition region and exclude the data as shown in Figure 5(b). Clearly, temperature and strain could be completely discriminated for the two separated sections.

## 4. Conclusions

A distributed OFS which can simultaneously measure temperature and strain has been demonstrated. BFS distribution is measured by using the DPP-BOTDA approach, while the RBSS is obtained through the OFDR technique. The distributed discrimination for temperature and strain can be achieved provided that a pair of BFS and RBSS along the sensing fiber has been measured. A 92 m long sensing range, a 50 cm spatial resolution with the temperature and strain accuracies of ±1.2 °C and ±15 με is achieved. The spatial resolution and measurable range could in principle be further improved.
